# Recent updates on daidzein against oxidative stress and cancer

**DOI:** 10.17179/excli2019-1847

**Published:** 2019-10-21

**Authors:** Sarita Rawat, Sachchidanand Pathak, Gaurav Gupta, Santosh Kumar Singh, Himmat Singh, Anurag Mishra, Ritu Gilhotra

**Affiliations:** 1 School of Pharmacy, Suresh Gyan Vihar University, Mahal Road, Jagatpura, Jaipur, India

## ⁯

***Dear Editor,***

Daidzein (7-hydroxy-3-(4-hydroxyphenyl)-4H-chromen-4-one) is a naturally occurring compound commonly found in soybeans and some other legumes. Daidzein is an isoflavone by nature and isolated from *Pueraria Mirifica*, having category of biologically active secondary metabolites commonly produced in the soybean growth and belong to the group of flavonoids. A number of pharmacological activities have been accounted for daidzein, which includes anti-carcinogenesis, anti-fibrotic, anti-diabetic, cholesterol-lowering and cardiovascular activity. Daidzein pretreatment was found to diminish the seriousness of mucosal damage in a portion subordinate way. Some other therapeutic uses of daidzein are weight reduction, decreasing bowels moment, and inflammation associated with histopathological deformities (Amaral et al., 2017[1]). Their structure and function are alike to human estrogen, which can play a massive role in the prevention of osteoporosis, cancer, and postmenopausal syndromes. Uses of daidzein in anticancer activity against ovarian cancer are still limited (Meng et al., 2017[14]). Epidemiological data suggest that increased utilization of soybean in food results in decreased cancer risk. Soybean food is highly recommended in cancer prevention because it has a number of anticarcinogens (Yu et al., 2017[23]). The potent antioxidant activity of daidzein reported in various *in vitro *and* in vivo *studies was conclusively shown in this literature (Table 1(Tab. 1); References in Table 1: Amaral et al., 2017[1]; Atiq et al., 2019[2]; Budryn et al., 2018[3]; Chan et al., 2018[4]; Davis et al., 2001[5]; Eskra et al., 2019[6]; Foti et al., 2005[7]; Gundogdu et al., 2018[8]; Hua et al., 2018[9]; Huang et al., 2019[10]; Karale and Kamath, 2017[11]; Liang et al., 2018[12]; Medeiros et al., 2016[13]; Meng et al., 2017[14]; Murata et al., 2004[15]; Park et al., 2016[16]; Poschner et al., 2017[17]; Rigalli et al., 2019[18]; Rohrdanz et al., 2002[19]; Sivoňová et al., 2019[20]; Uifalean et al., 2018[21]; Wei et al., 2019[22]; Zheng et al., 2017[25]; Zheng et al., 2018[24]; Zhu et al., 2018[26]).

EXCLI Journal 2019;18:950-954 – ISSN 1611-2156
Received: September 26, 2019, accepted: October 10, 2019, published: October 21, 2019

| Key findings | Reference |
|---|---|
| Daidzein could inhibit 5-FU-induced intestinal mucositis through prevention of relevant clinical symptoms, stimulation of the proliferative activity in the epithelium and crypts, suppression of oxidative stress and regulation of inflammatory homeostasis. | Atiq et al., 2019 |
| Soluble CXCL16 and VEGF-A are two promoters of angiogenesis and metastasis in breast cancer. The stimulation of these two angiogenesis-related cytokines might represent one of the mechanisms explaining the proliferative effects of low isoflavone doses in estrogen-dependent cells. In estrogen-independent cells, daidzein inhibited their secretion, indicating anti-angiogenic properties. | Uifalean et al., 2018 |
| The utilization of daidzein is associated with a reduced risk of prostate cancer but the direct effects of daidzein on the androgen receptor (AR) signaling pathway are not well understood. | Sivoňová et al., 2019 |
| Cytotoxic effects of daidzein on both MIA PaCa-2 and HT-29 cell lines occurred in a dose-dependent manner. Daidzein exhibits genotoxic potential under *in vitro* conditions. | Gundogdu et al., 2018 |
| Cell models and clinically possible concentrations of soy isoflavones do not support the notion that daidzein or other soy isoflavones can increase the effects of taxane chemotherapy in CRPC cell and xenograft models. | Eskra et al., 2019 |
| Genistein and daidzein inhibit the accumulation of lipid droplets (LDs) by regulating LDs-related factors and lead to final apoptosis of colon cancer cells and possible molecular mechanisms of isoflavones in anti-obesity and anti-tumor functions. | Liang et al., 2018 |
| Genistein, daidzein, and ERB-041 decreased ovarian cancer cell migration, invasion, proliferation, and bubble formation, signifying their roles on ovarian cancer cell metastasis, tumorigenesis, and their potential as other therapies for ovarian cancer patients. | Chan et al., 2018 |
| Daidzein exerted neuroprotective effect against ICV-STZ–induced behavioral parameters include cognitive deficits and locomotor impairment. Where daidzein was able to correct oxidative stress in neuronal cells in the brain. | Wei et al., 2019 |
| Daidzein enhanced cisplatin-impaired antioxidant defense such as reduced glutathione reserve, GPX activity, and SOD activity. | Meng et al., 2017 |
| Daidzein attenuates CP-induced oxidative stress on blood cells and antioxidants in rats. | Karale and Kamath, 2017 |
| Daidzein reduces hyperglycemia-induced oxidative stress in HUVECs. Thus, daidzein may be a potential therapeutic agent that could reduce hyperglycemia-induced oxidative damage associated with diabetes. | Park et al., 2016 |
| Daidzein dosing reduced the B cell population as well as IgG1 production, while increased the helper and cytotoxic T cell population in females. The shortage of protection or exacerbation of T1D can be explained by the reduced B cell population and increased regulatory cells, revealing a completely unusual mechanism compared to GEN exposure. | Huang et al., 2019 |

EXCLI Journal 2019;18:950-954 – ISSN 1611-2156
Received: September 26, 2019, accepted: October 10, 2019, published: October 21, 2019

| Key findings | Reference |
|---|---|
| Considering the activity of daidzein as nutraceutical that forms complexes with actin that are helpful in the prevention of cancer metastases. | Budryn et al., 2018 |
| Anti-proliferative action of 12 anticarcinogens from soybean against MCF-7 and MDA-MB-231 human breast cancer cell outline by single or two-way combination treatment. There is a synergistic result of the combination treatment of genistein plus daidzein MCF-7 cells. | Zhu et al., 2018 |
| Daidzein exerts similar activity against SKOV3 cells in nude mouse xenograft models and it was shown that daidzein considerably reduced the tumorigenesis in vivo, indicative of the potential for daidzein as a lead molecule in the growth of ovarian cancer chemotherapy. | Hua et al., 2018 |
| Daidzein may induce apoptosis of choriocarcinoma cells in a dose-dependent manner via the mitochondrial apoptotic pathway. | Zheng et al., 2018 |
| Daidzein and equol do not provoke a major up-regulation of the transporter expression but instead an inhibition of BCRP activity and sensitization to BCRP substrates. | Rigalli et al., 2019 |
| E1 and E2 conjugation by low micromolar concentrations of daidzein in the human breast cancer cell line MCF7, which leads to a small but statistically significant increase in unconjugated E2 of approximately 20 %. | Poschner et al., 2017 |
| The impact of the dietary isoflavone daidzein on the biochemical fingerprint of two human breast cancer cell lines – estrogen-responsive (MCF-7) and estrogen-unresponsive- was found to induce a noticeable decrease in cell growth. | Medeiros et al., 2016 |
| Daidzein is just as useful as genistein in protecting cells against oxidative damage especially with respect to DNA. Antioxidant activity of isoflavones could really contribute to the healthy properties of soy. | Foti et al., 2005 |
| Oxidative DNA damage by daidzein metabolites plays a role in tumor initiation and that cell proliferation by isoflavones via ER-ERE binding induces tumor support and/or progression, resulting in cancer of estrogen-sensitive organs. | Murata et al., 2004 |
| Antioxidant enzyme (AOE) system provoked by daidzein affected the oxidant rather than the antioxidant properties of daidzein. | Rohrdanz et al., 2002 |
| Daidzein supplementation may protect cells from oxidative stress-inducing agents by inhibiting NF-kappa B activation and decreasing DNA adduct levels. | Davis et al., 2001 |
| Daidzein can inhibit choriocarcinoma cell *proliferation in vitro* and *in vivo*; the underlying mechanism behind the inhibitory effects may probably be suppressing ERK pathway and afterward arresting cell cycle at G1 phase. | Zheng et al., 2017 |
| Biotransformation increased the anti-aromatase activity and the anti-tumoral value of daidzein in breast cancer cells. Moreover, it clarifies the potential use of daidzein in the prevention and/or treatment of ER+ breast cancer. | Amaral et al., 2017 |

## *Conflict of interest*

The authors declare no conflict of interest.

## Conflict of interest

The authors declare no conflict of interest.

## Figures and Tables

**Table 1:**
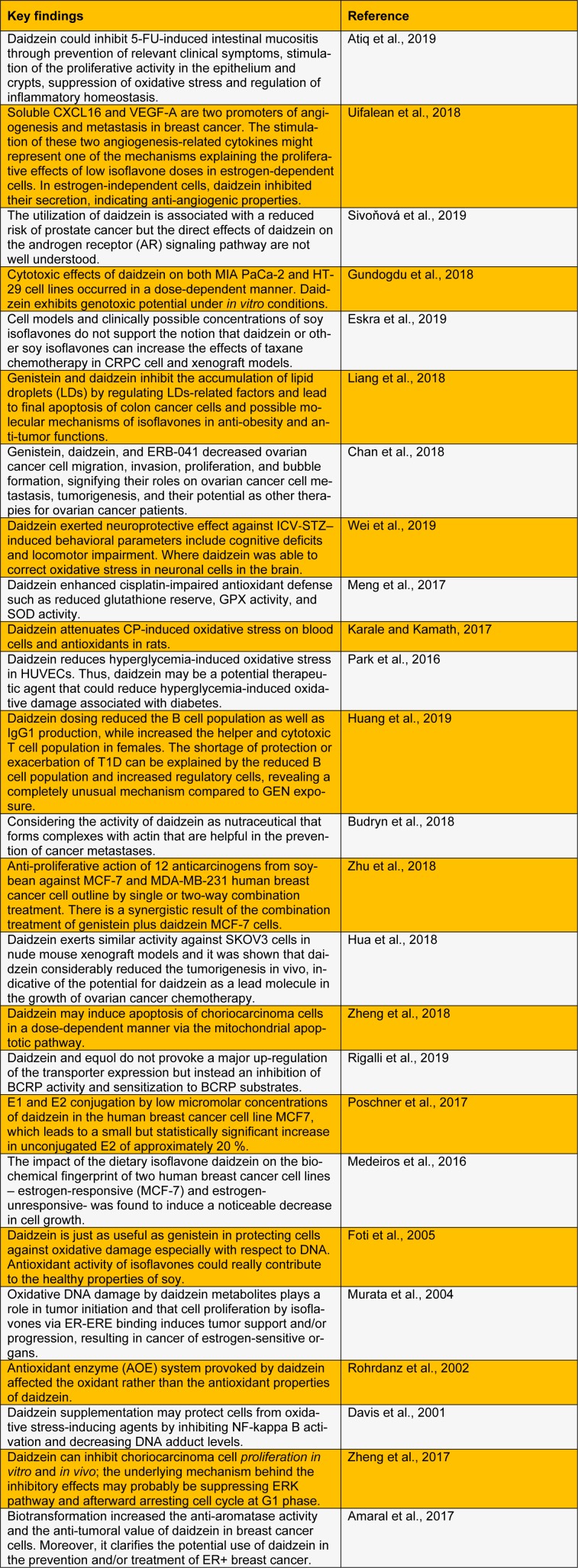
Recent updates on daidzein against oxidative stress and cancer
